# Un anévrysme sacciforme de l'aorte abdominale révélant une maladie de Behçet: quand faut-il opérer?

**DOI:** 10.11604/pamj.2014.19.252.5005

**Published:** 2014-11-07

**Authors:** Faida Ajili, Haifa Tounsi, Fatma Aouini, Najeh Bousetta, Nadia Ben Abdelhafidh, Bassem Louzir, Janet Laabidi, Salah Othmani

**Affiliations:** 1 Service de Médecine Interne, Hôpital Militaire de Tunis, 1008 Montfleury, Tunisie; 2 Service de Chirurgie Vasculaire et Unité de Greffe d'Organe, Hôpital Militaire de Tunis, 1008 Montfleury, Tunisie

**Keywords:** anévrysme artériel, maladie de Behcet, chirurgie, arterial aneurysm, Behcet disease, surgery

## Abstract

L'atteinte vasculaire est fréquente au cours de la maladie de Behçet. Elle est essentiellement représentée par les thromboses veineuses alors que l'atteinte artérielle est plus rare. Elle peut être isolée ou multifocale et peut toucher tous les territoires avec prédilection pour l'aorte abdominale, les artères pulmonaires et les artères des membres inférieurs. L'atteinte anévrysmale de l'aorte abdominale est trompeuse se manifestant par une symptomatologie atypique responsable d'un retard diagnostique favorisant la rupture. Dans notre cas, des douleurs abdominales paroxystiques ont incité le patient à consulter rapidement. L'enquête étiologique a conclue à un angio Behcet. Rarement, l'anévrysme de l'aorte abdominale est révélé par une complication telle que la rupture dans le rétro péritoine ou le tube digestif. Les indications chirurgicales des anévrysmes de l'aorte sont discutées ici. Un traitement immunosuppresseur au long cours s'impose en post opératoire pour limiter le risque de récidive.

## Introduction

La maladie de Behçet (MB) est une vascularite systémique d’étiologie inconnue, caractérisée cliniquement par une aphtose buccale ou le plus souvent buccogénitale associée à des manifestations systémiques dont les plus fréquentes sont cutanées, oculaires et articulaires et les plus graves sont neurologiques, cardio-vasculaires, intestinales et oculaires [1]. L′atteinte vasculaire est fréquente au cours de la maladie de Behçet. Elle est essentiellement représentée par les thromboses veineuses alors que l′atteinte artérielle est plus rare estimée de 2 à 8% des cas [2]. L′atteinte de l′aorte abdominale est trompeuse se manifestant par une symptomatologie atypique responsable d′un retard diagnostique [3]. Nous rapportons une observation d′un anévrysme de l′aorte abdominale sous rénale symptomatique révélant une maladie de Behçet.

## Patient et observation

Il s′agit d′un patient âgé de 21 ans, sans antécédents pathologiques notables, militaire de carrière, qui a consulté aux urgences pour des douleurs péri ombilicales paroxystiques associées à des vomissements post-prandiaux sans notion d′arrêt de matière ou de gaz ou de trouble du transit. A l'examen clinique, il était fébrile à 39° C et tachycarde à 100 b /mn. Les articulations étaient libres. L'examen cutanéo-muqueux avait objectivé 2 aphtes buccaux. L'examen neurologique était sans particularités. L'examen abdominal avait montré une masse abdominale battante. Une échographie couplée à une tomodensitométrie abdominale avait conclu au diagnostic d′anévrysme sacciforme de l′aorte abdominale sous-rénale. La biologie avait noté un syndrome inflammatoire biologique (CRP= 80mg/l, fibrinémie= 6). L'hémogramme avait montré une anémie normocytaire normochrome à 10 g/dl, une hyperleucocytose à prédominance de polynucléaires neutrophiles et des globules blancs à 120000 élt/mm3. Le taux des plaquettes étaient à 180000 élt/mm3. L'enquête infectieuse était négative (sérologie de wright, sérologie syphilis, hémocultures). L’échographie cardiaque n'a pas montré de signes d'endocardite infectieuse; ainsi un anévrysme d′origine mycotique a été écarté. Le diagnostic de maladie de Behçet a été retenu devant: une aphtose buccale récidivante (> 3 fois/ an), un test pathergique positif et l′angio- Behçet sous forme d′anévrysme de l′aorte abdominale. L'antigène HLA B51 était positif. L'examen ophtalmologique n'a pas montré de signes d'uvéite ou de vascularite rétinienne. Le patient a été traité par 3 boli de méthylprednisolone à la dose de 1 g/j pendant 3 jours de suite relayés par une corticothérapie orale à la dose de 1mg/kg/j associée au cyclophosphamide à la dose de 900 mg/ cure. Malgré ce traitement intensif, le contrôle scannographique a montré le même aspect sacciforme de l′anévrysme avec le risque de rupture, le patient a été alors opéré.

**Figure 1:**
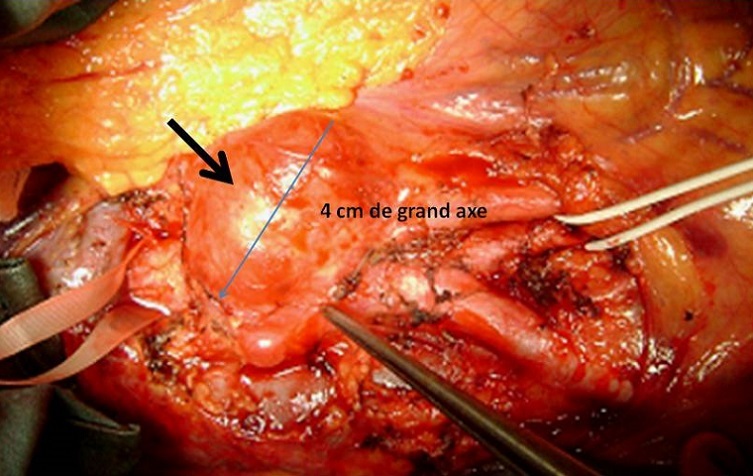
Vue per-opératoie d'un anévrysme sacciforme de l'aorte abdominale sous rénale chez notre patient

**Figure 2:**
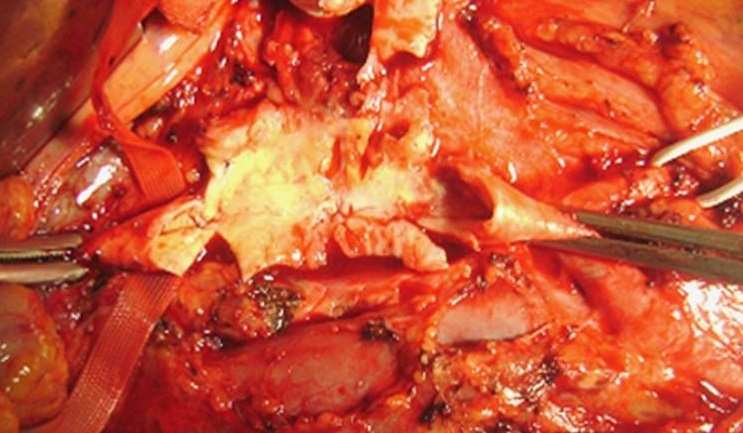
Mise à plat de l'anévrysme de l'aorte abdominal

**Figure 3:**
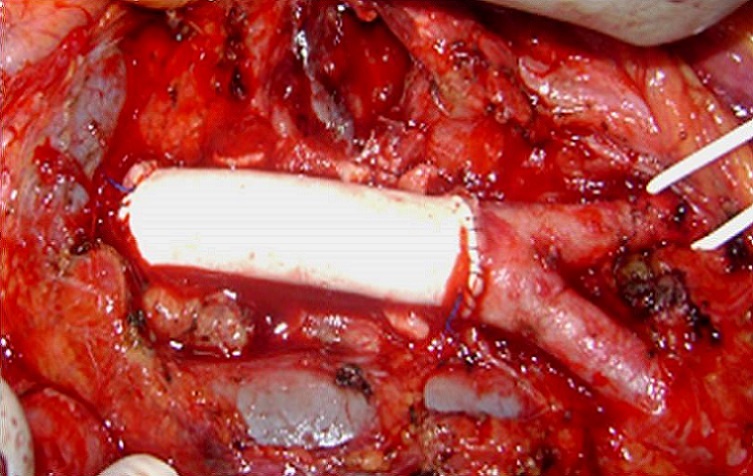
Greffe prothétique aorto-aortique en PTFE n° 12

En per-opératoire, on a noté la présence d′un anévrysme sacciforme latéral droit de 4 cm de grand axe partiellement thrombosé (Figure 1). Un contrôle aortique a été pratiqué en amont et en aval de l′anévrysme. Un clampage et une mise à plat de l′anévrysme ont été réalisés (Figure 2). Un tube en PTFE n° 12 a été interposé entre l′aorte sous rénale et la fourche aortique (Figure 3). Les suites opératoires ont été simples et le patient était gardé sous traitement corticoïdes à doses dégressives associée à la colchicine. L’évolution était marquée par la survenue d'un épisode de thrombose veineuse profonde secondaire à un arrêt intempestif du traitement corticoïde diagnostiqué un mois plu tard, lors de son admission pour le 2 bolus de cyclophosphamide. L’écho doppler avait montré une thrombose proximale fémoropoplité droite et le patient était remis sous traitement corticoïde associé à une héparinothérapie avec relais par les anti-vitamines k. Les anticoagulants ont été relayés par un antiagrégant plaquettaire au bout de 3 mois. Le cyclophosphamide prescrit pendant 6 mois a été relayé par de l'azathioprine à la dose de 150 mg/j. Le patient est actuellement sous colchicine (1cp/j), corticoïdes (5mg/j) et aspégic (100mg/j) sans récidive au niveau de l'anastomose ni ailleurs. Le recul est de 6 ans.

## Discussion

La maladie de Behçet est une vascularite systémique à tropisme surtout veineux. L′atteinte artérielle n'est présente que dans 2 à 8% des cas [2]. Elle revêt trois aspects; il peut s'agir de sténoses, de thrombose ou plus souvent d'anévrysmes artériels. Ceux-ci réalisent classiquement de véritables aphtes artériels qui correspondent à de véritable perforation à l'emporte pièce. Des lésions anévrysmales multiples étaient retrouvées chez ¼ des cas dans la série de 47 patients de Bensaid Y et al [4]. Depuis la publication du premier cas d'anévrysme aortique au cours d'une maladie de Behcet en 1961, le nombre de cas publiés ne cesse d'augmenter, vingt et un dans la série de Lakhanpal [5], cent trente et un dans la revue de littérature de Hamza [6].

Les AA peuvent intéresser tous les territoires avec une prédilection pour l′aorte abdominale, les artères pulmonaires et les artères des membres inférieurs [7, 8]. Ils surviennent souvent tardivement, cinq à dix ans après le début de la maladie, mais peuvent constituer le mode de révélation de la maladie comme se fut le cas chez notre patient [1]. Les manifestations cliniques dépendent du siège de l'anévrysme et de son stade évolutif. L′atteinte de l′aorte abdominale est souvent trompeuse réalisant une symptomatologie atypique responsable d′un retard diagnostique. D′autres causes de douleurs abdominales sont en effet évoquées plus souvent chez ces patients: iléite, thrombose veineuse profonde, complication d′un traitement immunosuppresseur. L′anévrysme de l′aorte abdominale peut rester asymptomatique et se révéler à l′occasion d′une complication telle qu′une rupture dans le rétro péritoine ou dans le tube digestif [9].

Dans notre observation, des douleurs péri-ombilicales paroxystiques ont amené le patient à consulter rapidement. L′enquête étiologique a conclu à un angio-Behçet. L'anévrysme de l'aorte abdominale est à rechercher en première intention par un échodoppler, examen non invasif capable de préciser le siège et la nature de la lésion artérielle [10]. L'artériographie artérielle a été déconseillée par de nombreux auteurs qui suggèrent que les ponctions artérielles peuvent induire la formation d'un faux anévrysme au point de ponction, comme ce fut le cas chez 3 malades dans la série de Ben Said et al [4]. L′angio-scanner et l'angio IRM sont devenues les examens de référence pour le diagnostic et le suivi de ces lésions artérielles [10]. Les anévrysmes sont responsables d′une mortalité voisine de 60% à cause de leur évolution rapide et inéluctable vers la rupture sans que la taille de l′anévrisme n′en soit considérée comme un facteur prédictif [2]. Ainsi dans le traitement de l'anévrysme artériel, la chirurgie ou l'approche endovasculaire sont généralement indiquées en raison du risque de rupture [11]. Cependant, Les indications chirurgicales ne sont pas clairement définies. Pour les anévrysmes de l′aorte abdominale, le traitement chirurgical fait le plus souvent appel à une mise à plat-greffe, anévrisectomie avec interposition d′un tube prothétique et implantation en zone macroscopiquement saine [12] comme c’était le cas chez notre patient. La chirurgie ouverte présente un taux de complication maximum de 50%, principalement sous forme de faux anévrysmes anastomotiques [13], mais aussi à type d'hémorragie peropératoire, lâchage anastomotique, fistule et occlusion du greffon.

La place du traitement endoluminal dans les anévrismes au cours de la maladie de Behçet, n′est pas bien codifiée. Il semble représenter une bonne alternative au traitement chirurgical, surtout quand la localisation rend difficile une éventuelle intervention chirurgicale ou quand le risque opératoire est élevé. Il devrait faire disparaître les faux anévrysmes anastomotiques [14]. Ces techniques exposent cependant au risque de développement d'anévrysme aux points de ponctions ou d'ancrage de la prothèse [14]. Dans la série de Kim et al, 20 anévrysmes ont été traités par endoprothèse et embolisation avec un taux de complication de 19% et de perméabilité primaire de 89% avec un recul de 24 mois [15]. L'association d'un traitement médical à base de corticostéroïdes et de cyclophosphamide visant à diminuer la récurrence de l′atteinte artérielle est recommandée par l'European League Against Rheumatism mais la durée du traitement immunosuppresseur après chirurgie n'est pas cosensuelle [16]. Le protocole le plus utilisé étant celui de boli de solumedrol relayés par une corticothérapie par voie orale à la dose de 1 mg/Kg/j associés à des boli mensuels de cyclophosphamide à la dose de 750 mg/m2/mois relayé par l'azathioprine. Wechsler [17] recommande un traitement par de l′azathioprine ou des boli de cyclophosphamide pendant douze à vingt-quatre mois dans les suites chirurgicales immédiates, puis un relais par colchicine et antiagrégants plaquettaires. Les anticoagulants au long cours ne sont pas uniformément prescrits hormis l′association à une thrombose veineuse [18] comme c’était le cas dans notre observation ou après une thrombose itérative de pontage [4]. Leur prescription ne doit pas être systématique, même en cas de pontage prothétique, dans la pathologie artérielle de la MB. Aucune publication à notre connaissance n′a démontré une plus grande efficacité des anti-vitamines K sur la perméabilité des pontages au long cours. En revanche, les risques hémorragiques sont majorés et les antivitamines K devraient à notre avis être proscrits dans la maladie anévrismale du Behçet [18].

## Conclusion

Devant un abdomen aigu chez un jeune patient, il faut toujours se méfier d'un anévrysme de l'aorte abdominale incitant à une recherche étiologique minutieuse.
